# Gut microbiota maturation during early human life induces enterocyte proliferation via microbial metabolites

**DOI:** 10.1186/s12866-020-01892-7

**Published:** 2020-07-11

**Authors:** Michael W. Dougherty, Oleksandr Kudin, Marcus Mühlbauer, Josef Neu, Raad Z. Gharaibeh, Christian Jobin

**Affiliations:** 1 Department of Medicine, Division of Gastroenterology, University of Florida, CGRC, 2033 Mowry Rd, Florida, 32610 USA; 2 Department of Pediatrics, University of Florida, Gainesville, Florida, USA; 3 Department of Infectious Diseases and Pathology, College of Veterinary Medicine, University of Florida, Gainesville, Florida, USA

**Keywords:** Bacteria, Intestine, Network, Epithelial cells, Stem cells, Bile acid, Metabolism

## Abstract

**Background:**

The intestinal tract undergoes a period of cellular maturation during early life, primarily characterized by the organization of epithelial cells into specialized crypt and villus structures. These processes are in part mediated by the acquisition of microbes. Infants delivered at term typically harbor a stable, low diversity microbiota characterized by an overrepresentation of various *Bacilli spp.*, while pre-term infants are colonized by an assortment of bacteria during the first several weeks after delivery. However, the functional effects of these changes on intestinal epithelium homeostasis and maturation remain unclear. To study these effects, human neonate feces were obtained from term and pre-term infants. Fecal 16S rDNA sequencing and global untargeted LC-MS were performed to characterize microbial composition and metabolites from each population. Murine enteral organoids (enteroids) were cultured with 0.22 μm filtered stool supernatant pooled from term or pre-term infants.

**Results:**

Term and pre-term microbial communities differed significantly from each other by principle components analysis (PCoA, PERMANOVA *p* < 0.001), with the pre-term microbiome characterized by increased OTU diversity (Wilcox test *p* < 0.01). Term communities were less diverse and dominated by Bacilli (81.54%). Pre-term stools had an increased abundance of vitamins, amino acid derivatives and unconjugated bile acids. Pathway analysis revealed a significant increase in multiple metabolic pathways in pre-term samples mapped to *E. coli* using the KEGG database related to the fermentation of various amino acids and vitamin biosynthesis. Enteroids cultured with supernatant from pre-term stools proliferated at a higher rate than those cultured with supernatant from term stools (cell viability: 207% vs. 147.7%, *p* < 0.01), grew larger (area: 81,189μm^2^ vs. 41,777μm^2^, *p* < 0.001), and bud at a higher rate (6.5 vs. 4, p < 0.01). Additionally, genes involved in stem cell proliferation were upregulated in pre-term stool treated enteroid cultures (*Lgr5, Ephb2, Ascl2 Sox9*) but not term stool treated enteroids.

**Conclusions:**

Our findings indicate that microbial metabolites from the more diverse gut microbiome associated with pre-term infants facilitate stem cell proliferation. Therefore, perturbations of the pre-term microbiota may impair intestinal homeostasis.

## Background

Shortly after birth, bacteria from the environment colonize the naïve gastrointestinal tract [1, 2]. In term infants, a low diversity microbiota harboring high proportions of *Bifidobacteria* (after vaginal birth) or *Staphylococcus* (after cesarean section) are typically observed shortly after delivery [3, 4]. In pre-term infants, microbiota maturation follows a ubiquitous pattern characterized by increased diversity and temporally distinct phases with an initial abundance of Bacilli followed by increased proportions of Gammaproteobacteria [5]. The characteristics of initial bacterial communities are closely linked to the maternal microbiome, suggesting colonization occurs during or shortly after birth with subsequent diversification resulting from environmental factors [6, 7].

This period of microbial acquisition represents a critical time in terms of development for the infant, and disruption of healthy microbiota acquisition may have acute or far-reaching pathological consequences such as necrotizing enterocolitis (NEC), late onset sepsis, and neurological disorders [8–11]. The presence of specific microbial species may have protective effects on the host. *Bifidobacteria* spp. maintain gut barrier function by preserving claudin 4 localization at tight junctions, and Clostridia are capable of modulating immune response to dietary antigens preventing allergic responses [12, 13]. Lactate synthesized by early colonizing microbes in the healthy neonatal intestine may serves as an energy resource for cells via recognition by Gpr81 receptors, subsequent oxidization to pyruvate, and TCA cycle incorporation [14, 15]. Lactate is also capable of promoting stem cell proliferation via Gpr81 receptors and increased WNT activity in adjacent Paneth cells [16].

In addition to the specific influence of particular microbes and associated metabolites, more diverse microbial communities may more efficiently fill functional niches independent of specific species composition. Gut bacteria enzymatically transform and metabolize undigested dietary components and host molecules consequently producing novel metabolites which, in part, mediate these effects [17]. Twin studies have shown microbial communities perform a core set of metabolic functions primarily providing sources of energy for the intestinal epithelium independent of differences in microbiota composition [18]. Bacteria ferment dietary carbohydrates and subsequently synthesize short chain fatty acids, which are preferentially utilized by the gut epithelium as source of energy and also have immunoregulatory functions [19]. A diverse array of common gut bacteria synthesize essential amino acids that serve as necessary precursors to cellular replication [20].

Given this evidence, we hypothesized that the distinct microbial communities acquired by term and pre-term neonates will generate distinct metabolomic profiles in the developing small intestine. In this study, we use small intestinal organoid models to quantify how microbial metabolites from each cohort affect proliferation and differentiation in the intestinal epithelium. We found that term infant microbial communities are characterized by a lack of diversity relative to pre-term infants. Fecal metabolomic profiles from these communities is also altered, with pre-term infant fecal metabolomics profiles characterized by increased signatures of amino acid metabolism, vitamin metabolism, and microbially transformed metabolites such as secondary bile acids. Soluble factors present in pre-term stool supernatants induce stem cell proliferation in organoid models. Taken together, this evidence suggests microbiota diversification in human neonates may facilitate intestinal proliferation.

## Results

### The pre-term infant gut microbiota is more diverse than the term gut microbiota

**Fig. 1:**
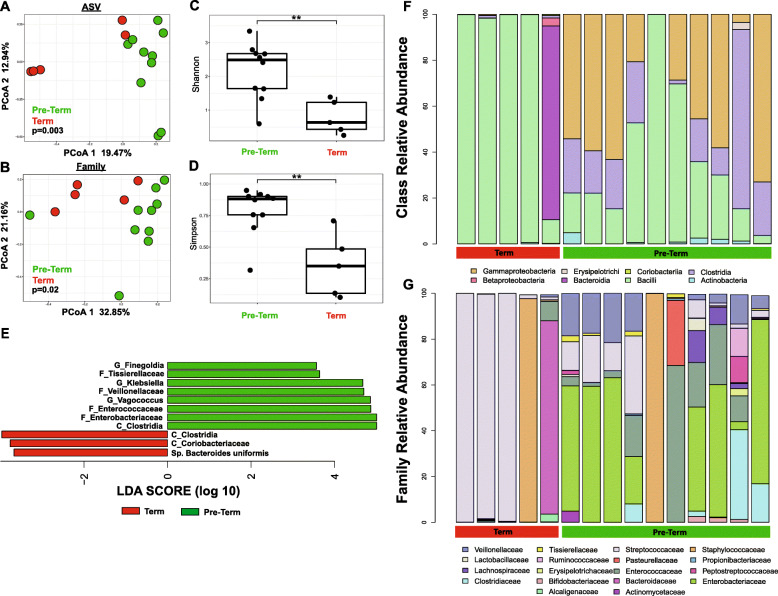
Pre-term and term infants harbor distinct gut microbiota. Principal Coordinates Analysis (PCoA) of term (*n* = 5) and pre-term (*n* = 10) microbial communities at (**a**) the ASV level (PERMANOVA *P* = 0.003) and (**b**) Family level (PERMANOVA *P* = 0.02). **c** Shannon (*n* = 15, Wilcoxon *P* = 0.008) and (**d**) Simpson communities (n = 15, Wilcoxon *p* = 0.008) diversity indices of term and pre-term communities. **e** Significantly enriched bacterial taxa in term and pre-term communities determined by Linear Discriminant Analysis Effect Size (LEfSe). Each bar represents a different ASV and only ASVs with *P*-value < 0.01 and LDA threshold value > 3.5 are shown. Microbiota composition in newborn and infant samples at the (**f**) class level and (**g**) family level

To study the interaction between host-bacteria associated with term and pre-term delivery, we first performed 16S rRNA gene sequencing to determine fecal microbial composition of neonates from each cohort. Two term samples showed very few 16S reads and were removed. Beta diversity analysis using principal coordinate analysis (PCoA) showed that bacterial communities differed significantly in their composition at both the ASV and family levels (Fig. 1a-b). Pre-term microbiome composition was characterized by increased alpha diversity (Shannon: *p* = 0.008, Simpson: p = 0.008; Fig. 1c-d) and beta diversity (*p* = 0.0084) compared to term composition.

Linear discriminant analysis was performed to determine the significantly enriched bacteria in each cohort. Pre-term communities were characterized by increased ASVs from various bacterial groups, including Clostridia, *Enterobacteriaceae*, Veillonellaceae, Enterococcaceae, Tissierellaceae, *Klebsiella*, *Vagococcus* and *Finegoldia* (Fig. 1e). Term infant microbial communities were dominated primarily by a single class of bacteria: Bacilli (4 of the 5 samples), or Bacteroidia (one of the 5 samples) (Fig. 1f). In pre-term infants, Gammaproteobacteria was the most common dominant taxa, followed by Clostridia and Bacilli (Fig. 1f). *Streptococcus* and *Staphylococcus* were the most abundant families in term stool samples (Fig. 1g). The pre-term microbiota was more diverse, with larger proportions of Enterobacteriaceae and Veillonellaceae, and lower relative abundances of early colonizers such as *Streptococcus* (Fig. 1g).

### The pre-term neonate metabolome is characterized by an increase in bacterially transformed products, vitamins, and amino acid derivatives

**Fig. 2:**
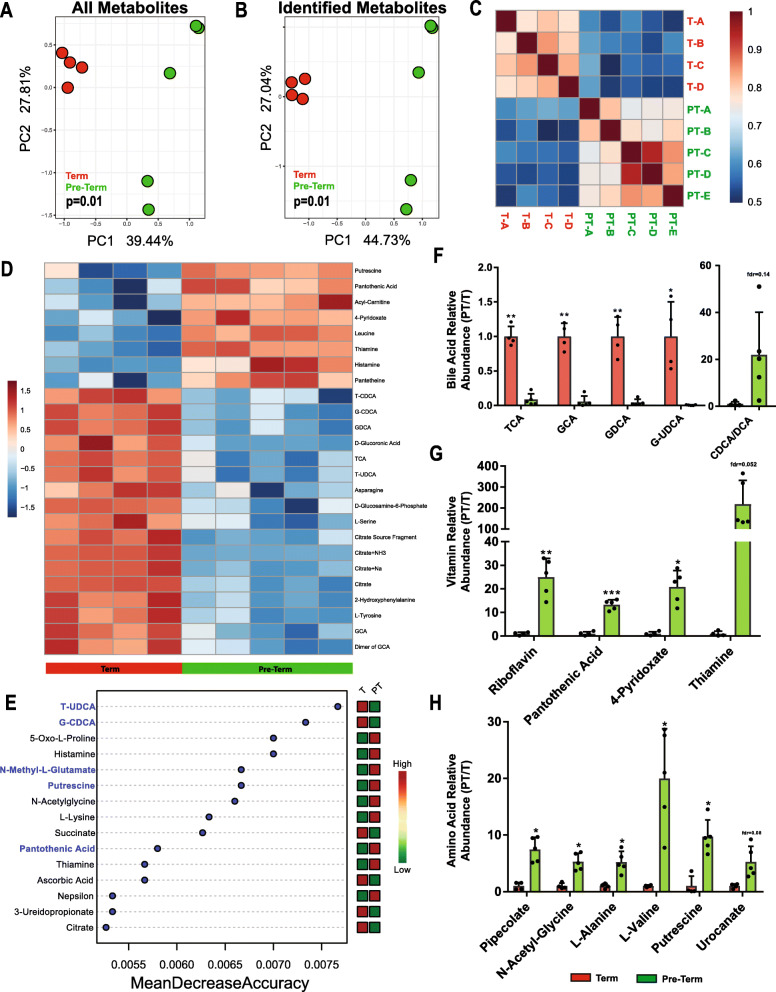
Distinct metabolomics profiles are associated with term and pre-term microbial communities. Principal component analysis (PCA) of term (*n* = 4) and pre-term (n = 5) infants listing (**a**) all metabolites (PC1 Wilcox *P* = 0.01) and (**b**) positively identified metabolites (PC1 Wilcox P = 0.01). **c** Heatmap visualizing correlations between identified metabolomics profiles. **d** Heatmap visualizing the top-25 most significantly enriched metabolites in term and pre-term metabolomics profiles. **e** Random forest identification of top 15 differentially abundant metabolites in term and pre-term metabolomics profiles, blue text identifies microbially synthesized or bio-transformed compounds. Difference in specific metabolite concentrations determined using Wilcoxon rank sum tests for (**f**) bile acids, (**g**) vitamins, and (**h**) amino acids. All *P* values are Benjamini-Hochberg corrected, * = fdr *P* < 0.05, ** = fdr *P* < 0.01. Metabolomics data is represented as the mean of three technical replicates. For all panels: T = term, PT = pre-term

To examine how these distinct microbial communities may functionally affect the host intestinal development, we performed global untargeted LC-MS and metabolomics on a subset of stools used in the 16S rRNA sequencing. A total of 14,342 metabolites were detected using positive and negative ion modes, and 374 metabolites were positively identified (Supplemental Tables 1–2), of which 48 metabolites were significantly altered between term and pre-term infants (Supplemental Table 3). Principal component analysis (PCA) showed significant differences in total metabolic composition (Fig. 2a) as well as composition of identified metabolites (Fig. 2b) with age at delivery. Similarly, metabolomics profiles of fecal samples from each cohort were highly correlated with each other (Fig. 2c).

Pre-term fecal samples exhibited metabolic profiles with a higher relative abundance of various vitamins, amino acids, or byproducts of amino-acid metabolism, while term fecal samples contained higher relative abundances of conjugated bile acids and intermediaries of the TCA cycle (Fig. 2d). Pre-term samples were classified by an increase in several bacterial-associated metabolites involved in amino acid metabolism (histamine, putrescine), bacterial-derived vitamins and associated enzymes (pantothenic acid, pantetheine), and a decrease in the primary bile acids glycodeoxycholic acid (GCDCA) and tauroursodeoxycholic acid (TUDCA; Fig. 2e). An increase in conjugated bile acids was observed in the term metabolome, concurrent with an increase in bacterially transformed bile acids in pre-term samples indicating an interaction between microbes and luminal host metabolites (Fig. 2f). However, metabolomics analysis was unable to differentiate between deoxycholic acid and chenodeoxycholic acid, and individual sample variation in these concentrations was high (Fig. 2f). Additionally, the pre-term metabolome had a higher relative abundance of various vitamins and amino acid derivatives (Fig. 2g-h).

These results show that increased microbiota abundance and diversity is associated with changes to overall fecal metabolomics profiles, and suggests that the microbiota may modulate molecules in the intestine by biotransformation of primary bile acids, fermentation of dietary amino acids, and vitamin biosynthesis.

### The neonatal intestinal microbiota influences gut metabolites

**Fig. 3:**
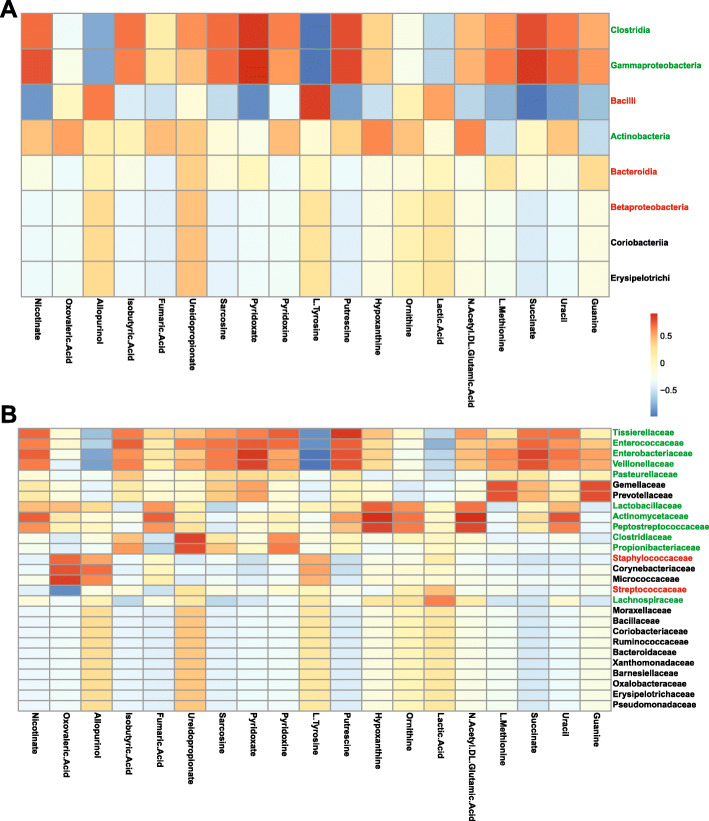
Correlation between known microbial metabolites linked to proliferation and microbiota composition. Heatmap visualizing Pearson’s correlation coefficients relating relative metabolite concentrations with relative bacterial abundance at (**a**) the class and (**b**) family level. Red text denotes bacterial taxa associated with term (n = 4) microbiota, green text denotes bacterial taxa associated with pre-term (n = 5) microbiota

Analysis at the class level showed the dominant classes from pre-term samples (Clostridia and Gammaproteobacteria) were correlated with an increase of several microbe-associated metabolites (Fig. 3a), while the dominant class from term samples (Bacilli) correlated with a decrease of several microbe-associated metabolites (Fig. 3a). Higher abundance in three pre-term enriched families (Enterobacteriaceae, Enterococcaceae, and Tissierellacea) correlated with an increase of several microbially associated metabolites implicated in promoting proliferation in organoid models [21] (e.g. nicotinate, sarcosine, pyridoxate/pyridoxamine, succinate, uracil; Fig. 3b). Additionally, an increase in metabolites related to proliferation via amino acid synthesis or metabolism were correlated with these bacterial families (e.g. Ureidopropionate; Fig. 3b). Taken together, these data suggest that potential functional changes occur as the microbiome diversifies during early life, possibly through biosynthesis of bacterial metabolites or modulation of dietary/host-derived metabolites such as primary bile acids.

**Fig. 4:**
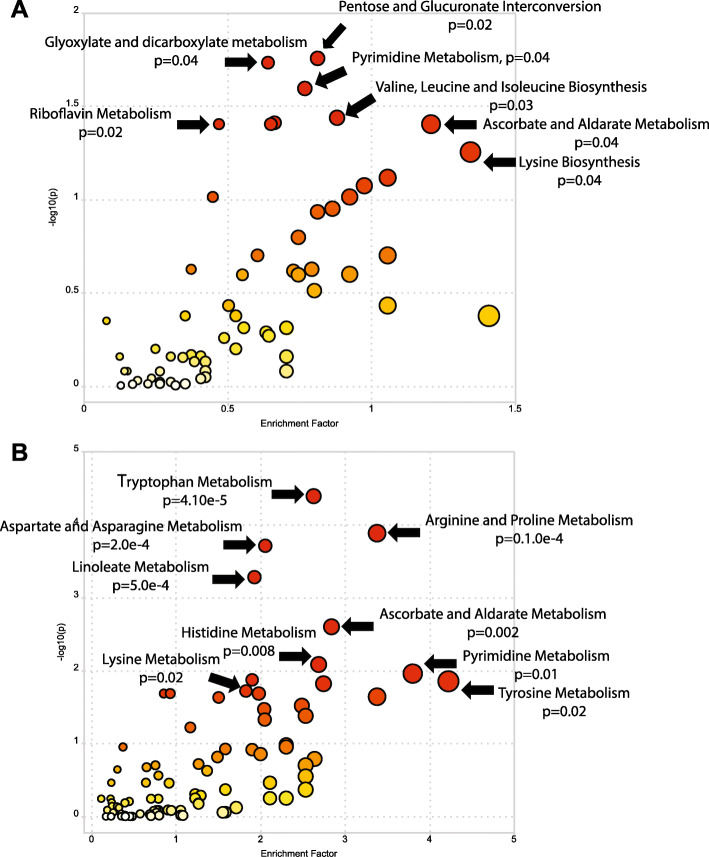
KEGG metabolomic pathway analysis showing significantly enriched pathways in pre-term infant cohort. Metabolic pathways enriched in PT samples mapped to *Homo sapiens* (**a**) and *Escherichia coli* (**b**), with the most significantly enriched pathways identified; *P* values under each pathway’s name denote enrichment of entire pathway by Fisher’s exact test

In order to understand how bacterial/host biosynthesis or transformation of metabolites shaped the metabolome, we identified enriched metabolic pathways in infant samples using KEGG (Kyoto Encyclopedia of Genes and Genomes) pathways of the model bacteria *Escherichia coli* and the human host, *Homo sapiens*. When metabolomics data was mapped to *E. coli*, a significant increase in 8 pathways was detected in pre-term neonates (Fig. 4a). Upregulated pathways in these microbial communities primarily related to amino acid metabolism, and biosynthesis of essential vitamins (e.g. pantothenic acid) and amino acids (e.g. lysine) (Supplemental Table 4). When mapped to *H. sapiens*, 20 significantly enriched pathways predominately related to the metabolism of various amino acids and vitamins (Fig. 4b, Supplemental Table 5). Degradation of the essential amino acid lysine was increased in conjunction with an increase in lysine biosynthesis by bacteria, demonstrating potential host utilization of microbial metabolites (Fig. 4a-b). Similarly, upregulation of human histidine metabolism was observed in pre-term samples, likely accounting for increased urocanate (a derivative of l-histidine) concentrations (Supplemental Table 5).

### Metabolites from pre-term microbial communities increase enterocyte proliferation

**Fig. 5:**
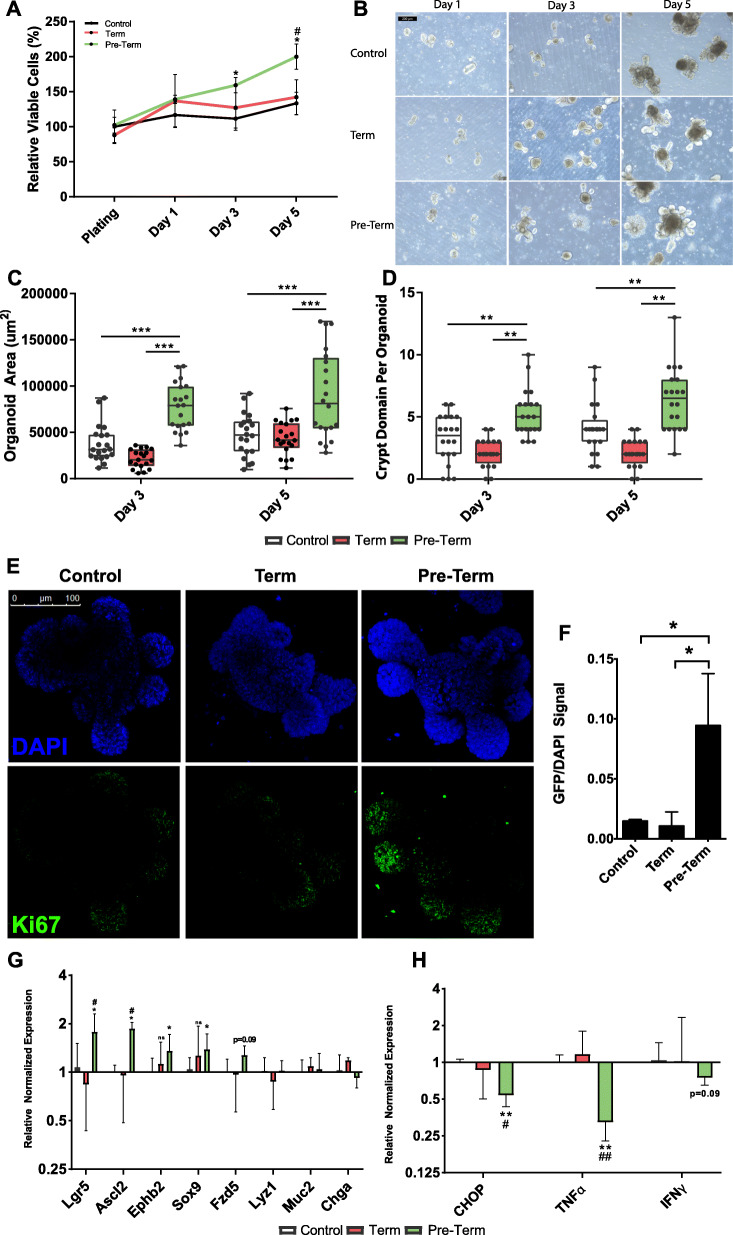
Enteroid culture with pre-term metabolites, but not term metabolites, increases proliferation by intestinal stem cell expansion. **a** MTT assay quantifying enteroid proliferation in presence of term and pre-term metabolites (*n* = 9 wells per data point pooled from 3 biological replicates). **b** Representative images showing enteroids cultured with term and pre-term metabolites. **c** Enteroid area after 3 and 5 days culture with term and pre-term metabolites (*n* = 20 per group pooled from 2 biological replicates). **d** Number of crypt domains per enteroid after 3 and 5 days culture with term and pre-term metabolites (n = 20 per group pooled from 2 biological replicates). **e** Ki67 immunofluorescence in enteroids cultured with term and pre-term stool supernatant. **f** Quantification of relative Ki67/DAPI signal area in stool supernatant treated enteroids (*n* = 3 images per group). **g** Quantitative RT-PCR analysis for markers of proliferation and differentiation in term and pre-term treated enteroids and (**h**) markers of ER stress and inflammation in term and pre-term treated enteroids (*n* = 6 per sample pooled from 2 biological replicates). *P* values calculated using unpaired t-test or Mann-Whitney U tests for all experiments. For all tests, * = P < 0.05, ** = P < 0.01, *** = *P* < 0.001, control vs. pre-term. # = P < 0.05, ## = P < 0.01, term vs. pre-term

To examine the potential effects of neonatal gut metabolites on enterocyte development, murine enteroids were cultured with pooled filtered stool supernatants obtained from human term and pre-term neonates previously analyzed by metabolomics. Cellular proliferation was measured by MTT assay, and results showed an increase in cell abundance after 3- and 5-days treatment with pre-term stool supernatans (Fig. 5a). Enteroids cultured with pre-term stool supernatant grew more rapidly than term or control enteroid cultures, exhibiting a more robust phenotype after 5-days of culture indicating more efficient growth and maturation (Fig. 5b-d). Additionally, enteroids treated with pre-term stool supernatants formed crypt domains earlier and at a higher rate than term-supernatant and control treated enteroids (Fig. 5d). Enteroids cultured with pre-term stool supernatant also exhibited increased Ki67 localized primarily to the base of newly budding crypts (Fig. 5e,f). Collectively, these data suggest that metabolites present in stool samples harboring more abundant and diverse microbiota induce enterocyte proliferation.

In addition, we observed increased *Lgr5*, *Ascl2*, and *Ephb2* mRNA expression in pre-term treated enteroids, which are markers of stem cell proliferation, and concomitant increase in Wnt signaling genes *Sox9* and *Fzd5* [22] (Fig. 5g). No difference was observed in markers of differentiation towards other enterocyte lineages (*Lyz1*, *Muc2*, *Chga*). Pre-term stool supernatant treatment also slightly downregulated markers of ER stress and inflammation (*CHOP, TNFa,* IFNy; Fig. 5h). Taken together, this evidence suggests that the proliferative phenotype is driven by stem cell niche expansion in response to microbial metabolites isolated from neonate stool harboring a diverse microbiome.

## Discussion

Establishment of a healthy microbiome in infants is essential for normal growth and development of intestine, optimal metabolic function as well as maturation of the immune system [17, 23, 24]. Therefore, altered microbiota composition puts infants at risk for both short- and long-term health implications. In healthy infants, the intestine is typically colonized by lactic acid bacteria (LAB) derived from breast milk [25]. Enteroids cultured with *Lactobacillus* exhibit increased Ki67 expression, and adding lactate to the culture increases stem cell proliferation by increasing WNT production in adjacent Paneth cells [16, 26]. Given the increased LAB abundance in the term composition we expected to observe a difference in lactate level between our samples and increased epithelial proliferation in enteroids treated with term stool supernatants [16]. Interestingly, lactate levels between term and pre-term samples were not different, possibly because the host utilizes these metabolites prior to excretion, and no increased proliferation was observed. Because of this, the lack of a proliferative phenotype in enteroids treated with term infant stool supernatants may suggest that intestinal maturation in healthy infants is driven by key microbes and metabolites missing in our stool supernatants rather than the robust function of a complex community.

Alternatively, epithelial proliferation may increase through the total metabolic output of a complex microbial community which more effectively shapes the total metabolomics profile. Several studies have shown that mice harboring a complex microbiota exhibit higher rates of proliferation and tissue growth than germ free counterparts, a phenomenon which may result from editing of metabolomic profiles by the microbiota [27, 28]. Gut microbes perform a set of core metabolic functions [18], pathways related to core microbial metabolic functions (pyrimidine metabolism, amino acid metabolism, and the biosynthesis of secondary metabolites) were significantly upregulated in our pre-term supernatant samples. Treatment with pre-term stool supernatant increased organoid proliferation accordingly, suggesting soluble factors in the stool mediate these effects. Increased absolute cell numbers demonstrate that these changes derive from increased cell cycling, rather than increased luminal contents or cell distension. Furthermore, mRNA expression profiles are consistent with increased stem cell proliferation and WNT signaling pathways. Notably, organoids increased in size and crypt domains rather than forming “cystic” structures typical of undifferentiated stem cells. One could hypothesize that the proliferative effect of pre-term gut metabolites observed in our study may serve a protective role for the epithelium. Perturbations of these communities (e.g. exposure to antibiotics) and concurrent dysbiosis may limit these effects and put pre-term infants at risk for disease.

Determining which specific metabolites and host-microbe dynamics from our pre-term stool supernatants increase proliferation may have therapeutic potential. In our study, the more diverse pre-term microbiota was characterized by metabolomics profiles with a significant increase of amino acid derivatives, such as putrescine. Putrescine is a polyamine, a class of molecules which serve as a precursor to cellular division whose concentrations are particularly high in rapidly proliferating tissue such as the small intestine [29, 30]. Putrescine is involved in nucleic acid and protein synthesis during cellular proliferation, preceded by the generation of putrescine by decarboxylation amino acids such as arginine and ornithine [31, 32]. Putrescine itself is synthesized by a number of bacterial species and has been shown to slightly increase proliferation in organoids [21, 33, 34]. In our study, both ornithine and putrescine abundances correlated to specific bacterial groups characterizing the pre-term microbiome including Clostridia, Gammaproteobacteria, and Actinobacteria. Additionally, urocanate and histamine were more abundant in pre-term samples, coupled with increased histidine metabolism. Urocanate and histamine are both byproducts of l-histidine metabolism, and l-histidine is an essential amino acid biosynthesized by many bacterial species [35]. L-histidine deficiency has been shown to induce mitochondrial dysfunction in the intestinal epithelium, and histamine receptor agonists increase epithelial proliferation in the gut [36, 37]. The increased urocanate and histamine concentrations in pre-term metabolomics profiles suggest increased deamination of l-histidine in the presence of a complex microbiota, a phenomenon that may enhance epithelial viability and mitochondrial function. Taken together, this evidence suggests the microbiota may supply amino acids necessary for cellular proliferation, which may increase stem cell cycling and viability rate. However, formula and breast milk generally contain amino acids and thus it remains unclear if amino acid biosynthesis by bacteria is an essential component of development or whether the host receives adequate quantities of essential metabolites from the diet.

Bile acid deconjugation was significantly increased in the pre-term metabolomes, yielding high deoxycholic acid (DCA) concentrations. Lower concentrations of primary bile acids in pre-term samples were coupled with an increase in primary bile acid biosynthesis suggesting that pre-term microbial communities are efficiently biotransforming host products to DCA [38]. A large majority of bile acids are reabsorbed by the ileum, and DCA is of particular interest due to its hydrophobic nature and increased permeability through the small intestine [39]. Once reabsorbed, bile acid derivatives play a key role in modulating microbial communities by acting on Farnesoid X receptors (FXRs) present on multiple epithelial lineages including Lgr5+ stem cells [40, 41]. Recent evidence suggests FXR activation regulates proliferation by inhibiting stem cell expansion, and that DCA exposure counters this inhibition by blocking FXR gene targets and activating EGFR signaling pathways [42–45]. EGFR signaling is a crucial component of stem cell niche maintenance in the gut. EGF and EGF-like growth factors have been shown to increase intestinal stem cell proliferation and protect against anoxia induced stem cell apoptosis and bacterial translocation in both mouse models and cell lines [46]. Furthermore, organoid models exposed to DCA exhibit increased expression of WNT pathway genes and stem cell markers [42]. During wound healing, crypt DCA concentrations greatly increase as FXR activation facilitates stem cell regeneration by inhibiting PGE2 expression, and DCA has been correlated with sustained remission in pediatric Crohn’s disease [47, 48]. Taken together, these findings suggest that bacterial transformation of conjugated cholic acids to DCA may play a protective role in the developing pre-term gut, driving stem cell proliferation during early life. More work is necessary to determine how various concentrations and temporal patterns of DCA exposure may regulate intestinal maturation throughout early life.

Epithelial maturation facilitated by microbial colonization has been well defined in preclinical mouse models. Here we provide evidence that similar phenomenon occur in human neonates during early life. Functionally, increased proliferation and tissue maturation is an essential developmental process particularly in pre-term infants, and increased proliferation can be beneficial during early life as a method of overcoming stem cell disfunction resulting from endoplasmic recticulum stress [49] or mitochondrial dysfunction [50]. Similarly, microbially-induced stem cell proliferation supports appropriate crypt-villus architecture and formation of deeper progenitor zones which may support epithelial health by protecting intestinal stem cells from microbial interaction [21, 27].

A limitation of our analyses is the inability to decouple microbial and host-derived metabolites, which we can only achieve through a priori knowledge. Furthermore, untargeted metabolomics is limited by the available metabolite library and generally only provides a small number of confirmed metabolite identities. Thus, identity limitations may obfuscate metabolites inducing proliferative phenotypes. Metabolite screening by Kaiko et al. has shown a large number of microbial metabolites, including some discussed here, only slightly enhance organoid proliferation when monoassociated in vitro [21]*.* Given the more robust proliferation response we observed, this suggests there may be an additive or complementary effects when considering the functional effects of entire microbial communities. Additionally, specific metabolites present in the term gut may not be present in fecal samples or in the processed stool supernatant, thus it may not be possible to conclude that microbiota-induced proliferation is strictly limited to pre-term bacterial communities. For example, microbial communities in breast-fed term infants are typically characterized by a long period of low diversity concurrent with increases in overall bacterial abundance and normal epithelial maturation. Thus, this work primarily highlights the potential for the pre-term gut microbiota to induce proliferation and the potential detrimental effects stemming from dysbiosis during this critical window.

## Conclusions

The evidence presented here demonstrates the proliferative effect of pre-term metabolomes on small intestinal organoids. We employ metabolomics analysis to gain insight into potential regulators of proliferation during early life, laying a foundation for further research analyzing the role of microbially derived metabolites in developmental processes. While many of the individual compounds highlighted here have been shown to have marginally proliferative effects on epithelial organoids, our study suggests potential additive effects derived from entire microbial communities. Future studies should focus on defining the functional impact of metabolic pathways outlined here in pathological situation such as necrotizing enterocolitis.

## Methods

### Preparation of stool supernatant

Stool samples were collected from human infants from the neonatal intensive care unit (NICU) and the newborn nursery. Term samples (*n* = 7) were collected from term stool/meconium and pre-term samples (*n* = 10) were collected from pre-term stool. Pre-term samples were obtained from infants undergoing routine care in the NICU, often characterized by compulsory antibiotic treatment during the first several days of life. Samples were stored briefly at − 20 °C before being transferred on dry ice and placed at − 80 °C for long-term storage. Stool samples (1 g) were suspended in sterile PBS, dissociated by pipetting with a wide bore tip and vortexed vigorously for 30 s then centrifuged and the supernatant was passed through a 0.22 μm filter before storage at − 80 °C.

### 16S rRNA gene sequencing

DNA was extracted from neonate stool samples (term: n = 7, pre-term: n = 10) using the Powerlyzer Powersoil DNA isolation kit (Qiagen 12855) according to manufacturer’s specifications with slight modifications. The V1-V3 hypervariable region of the 16S rRNA gene was amplified using primer pair 8F (5′- AGAGTTTGATCCTGGCTCAG − 3′) and 534R (5′-ATTACCGCGGCTGCTGG-3′) as previously described [51]. Briefly, forward and reverse primers contained universal Illumina paired-end adapter sequences with unique barcodes between the PCR primer sequence and the Illumina adapter sequence to allow multiplex sequencing. Equimolar amounts of samples were then pooled and sequenced with an Illumina MiSeq.

### Analysis of 16S rRNA sequences

Sequencing reads were preprocessed using Quantitative Insights into Microbial Ecology version 2 (QIIME2) [52] including trimming, filtering at Q20 and pair merging. The final set of reads was fed to the DADA2 algorithm within QIIME2 pipeline to infer exact amplicon sequence variants (ASVs) with trim length set to 200 (the average sequence length of the dataset) [53]. Two newborn samples showed very few read count (less than 20 reads) and therefore were removed from subsequent analyses. The remaining samples contained an average of 51,885 reads per sample (min = 33,516 reads; max = 81,138 reads) incorporated in ASVs. Taxonomic assignment was done using QIIME2’s feature-classifier classify-sklearn after training the classifier on the Greengenes 97% reference dataset (release 13_8). Only ASVs with bacterial taxonomy were used in subsequent analyses.

We generated Principal Coordinate Analysis (PCoA) using the phyloseq R package [54] from Bray-Curtis dissimilarity matrix after count normalization and log_10_ transformation using the following formula [55]:

$$ {\mathit{\log}}_{10}\left(\frac{RC}{n}\ \mathrm{x}\ \frac{\sum x}{N}+1\right) $$

where *RC* is the read count for a particular ASV in a particular sample, *n* is the total number of reads in that sample, the sum of *x* is the total number of reads in all samples and *N* is the total number of samples. Family PCoA (Fig. 1b) was generated as described above except that ASVs were merged at the family level. For Fig. 1f and g, only taxa with a relative abundance > 1% are shown.

Difference in the microbial community composition (beta diversity) was tested using permutational multivariate analysis of variance (PERMANOVA) through the vegan R package command adonis (version 2.5) with permutations set to 1000.

Alpha diversity (Shannon and Simpson indices) was calculated using the phyloseq R package from the rarefied counts (samples rarefied to the minimum count in all samples: 33,516) and differences were tested using Wilcoxon rank sum test using R function wilcox.test.

Linear discriminant analysis effect size (LEfSe) [56] was used to identify biomarkers associated with Phase1 and Phase2 and only OTUs with a *P*-value < 0.01 and LDA threshold value > 3 were considered a significant association.

### Metabolomics extraction and LC-MS

A subset of neonate stool samples with additional available fecal material (term: *n* = 4, pre-term: *n* = 5) were used for further metabolomics analysis. Samples were suspended in 200 μL 5 mM Ammonium Acetate and homogenized thrice for 30 s each time using a cell disruptor. Protein concentrations of the homogenates were measured using a Qubit fluorometer and normalized to 500 μg/mL protein. Five microliter of internal standard mixture was spiked into each normalized sample. Extraction of metabolites was performed by protein precipitation by adding 200 μL of extraction solution consisting of 8:1:1 Acetonitrile: Methanol: Acetone. Samples were mixed thoroughly, incubated at 4 °C to allow protein precipitation, and centrifuged at 20,000 g. Supernatant was transferred into a new tube and dried using nitrogen. Samples were reconstituted with 25 μL of reconstitution solution, mixed, and incubated at 4 °C for 10–15 min. Samples were centrifuged at 20000×g and supernatants collected for metabolomics profiling.

Global metabolomics profiling was performed on a Thermo Q-Exactive Orbitrap mass spectrometer with Dionex UHPLC and autosampler. All samples were analyzed in positive and negative heated electrospray ionization with a mass resolution of 35,000 at m/z 200 as separate injections. Separation was achieved on an ACE 18-pfp 100 × 2.1 mm, 2 μm column with mobile phase A as 0.1% formic acid in water and mobile phase B as acetonitrile. The flow rate was 350 μL/min with a column temperature of 25 °C. Four microliter was injected for negative ions and 2 μL for positive ions. Three technical replicates were performed per sample.

### Metabolomics analysis

The mean of the three technical replicates was generated for each sample and used for analyses. Data were normalized by sum and log-transformed prior to analysis. Principal component analysis (PCA) was done using R prcomp function and difference between the two groups at PC1 was tested using Wilcoxon rank sum test using R function wilcox.test and *p*-values corrected for multiple comparisons using R function p.adjust using the method of Benjamini and Hochberg [57]. Data was further analyzed using Metaboanalyst software [58]. Only identified metabolites were included in these analyses. Metabolite analysis was conducted using the suggested mass tolerance of 0.25 m/z and retention time of 30 s, and filtered by interquartile range. Random forest was used to determine important metabolomics features using 500 trees. KEGG pathway analysis was conducted using the Metaboanalyst MS Peaks to Pathways module with a p-value cutoff of 0.01. Pathway enrichment was determined by adjusting normalized metabolite concentrations from pre-term samples relative to term samples, and significance assessed by Fisher’s exact test.

We cross-referenced normalized identified metabolite concentrations from our samples with a list of known microbially-associated metabolites obtained from Kaiko et al. [21] and correlated their relative abundances with 16S rRNA sequencing data from the same samples. Pearson correlation coefficients were calculated using the R function cor.test and heatmaps generated using the R package pheatmap [59].

### Organoid preparation

Small intestinal enteroids were generated from 4 to 8 week old wild type C57BL6 mice as previously described [51]. Enteroids were cultured in Advanced DMEM/F12 (Life Technologies 12,634) supplemented with 1x N-2 supplement (R&D Systems AR009), 1x B27 supplement (Gibco 17,504), 10 mM HEPES (Thermo Fisher Scientific15630080), 1x Glutamax (Gibco 15,630,080), 100 U/mL Penicillin-Streptomycin (Gibco 10,378,016), 50 ng/mL recombinant mouse EGF (Peprotech 315–09), 50 ng/mL recombinant murine noggin (Peprotech 250–38), and 50 ng/mL recombinant mouse r-spondin CHO-expressed (R&D Systems 7150-RS). Organoid cultures were passaged a minimum of one time prior to experimentation.

### Organoid proliferation

Organoids were seeded in 10 μl matrigel (Fisher Scientific 356,237) to 96-well culture plates at a density of approximately 40 organoids per well and exposed to the pooled term (*n* = 4) and pre-term (*n* = 5) stool supernatant from the same samples used for metabolomics analysis. Supernatants were diluted at a 1:100 dilution for up to 5 days with daily media changes. Viability assays were conducted using the CellTiter 96 Non-Radioactive Cell Proliferation Assay (Promega G4000) and absorbance was measured using a BioTek Synergy H4 Hybrid Microplate Reader. Measurements were normalized to mean absorbance values at plating to quantify changes in cell numbers. To measure organoid budding and area, approximately organoids (200 in 80 μl matrigel) were seeded to 6-well culture plates and treated with stool samples at a 1:100 dilution for up to 5 days. A minimum of 5 representative images were taken per treatment, and a researcher blinded to experimental treatment used a random ROI generator to select organoids for morphological analysis using ImageJ.

### Immunofluorescent staining

Mature, budding enteroids were passaged as previously described and treated by addition of stool supernatants at a 1:100 dilution for 3 days directly after passage. Organoids were collected, fixed, and permabilized as previously described [51] before incubation overnight at 4 °C with 0.5 μg/ml Ki67 antibody (Abcam #ab15580), then for 1 h with Alexa Fluor 488 goat antirabbit secondary antibodies (Life Technologies #A-11034). Specimens were then counterstained with 1:5000 DAPI solution (ThermoFisher #62248) for 1 h, mounted with Vectashield hard set mounting medium (Vector Laboratories #H-1400) in a fluorodish (World Precision Instruments FD35–100) and imaged using a Leica TCS SP5 confocal microscope. Fluorescence was quantified using ImageJ to compare the total area of GFP stain relative to DAPI.

### Quantitative RT-PCR

For qPCR analysis, enteroids were cultured in 6-well cultures plates as described above, treated for 48 h with stool supernatant, then collected and washed in cold PBS before resuspension in RLT buffer (Qiagen 217,004). RNA was isolated from two wells using the RNeasy Miniprep Kit (Qiagen 217,004) and RT-PCR was performed using the iScript cDNA Synthesis Kit (Bio-Rad 170–8891). qPCR was performed using a Bio-RAD CFX384 Real Time PCR system using primers described in Table 1. RT-PCR was performed for 35 cycles with a Tm of 56 °C and ΔCt values were calculated using the housekeeping gene β-actin. Fold change was calculated using the -2^ΔΔCt^ method [60].

**Table 1:** Quantitative RT-PCR Primer Sequences

Gene	5′-3′ Forward Primer	3′-5′ Reverse Primer
Lgr5	GCTTTGACACACATTCC	CTTCTCAAATTCTGTAGCG
Ascl2	AAGCACACCTTGACTGGTACG	AAGTGGACGTTTGCACCTTCA
Ephb2	GCGGCTACGACGAGAACAT	GGCTAAGTCAAAATCAGCCTCA
Sox9	CATCTCTCCTAATGCTATCTTC	CTGAGATTGCCCAGAGT
Fzd5	CACGGTTGTCTTCCTCTTAGTC	ACCTGCGATGGCTTCATT
Lyz1	GCCGATACTGGTGTAATG	CTCTCACCACCCTCTTT
Muc2	CCATCTCTACCACCATTAC	CTCGATCACCACCATTT
Chga	GTCCTGGAAGTCATCTC	AGTTCCTTCAGCAGATT
Chop	AGGAAACGAAGAGGAAGAA	CTGACTGGAATCTGGAGAG
TNF-α	ATGAGCACAGAAAGCATGATC	TACAGGCTTGTCACTCGAATT
IFNγ	ACGCTTATGTTGTTGCTGATGG	CTTCCTCATGGCTGTTTCTGG
β-actin	TGGCGTGAGGGAGAGXXXAGX	AGCCATGTACGTAGCCATCCA

### Statistics

For organoid experiments, all statistical tests were performed using GraphPad Prism (version 6). The rest of the analyses were done using R [61] (version 3.4.4). All tests were two-tailed and determined statistically significant at *p* < 0.05. Parametric tests were used to analyze normally distributed data, Mann-Whitney U tests were used for all other data. For all enteroid experiments biological replication was achieved by pooling results from organoids isolated from unique mice.

## Supplementary information

**Additional file 1: Supplemental Table 1.** LC-MS data collected in positive ion mode. Raw data from term and pre-term LC-MS analysis including mass, retention time, and positive metabolite identification where available.

**Additional file 2: Supplemental Table 2.** LC-MS data collected in negative ion mode. Raw data from term and pre-term LC-MS analysis including mass, retention time, and positive metabolite identification where available.

**Additional file 3: Supplemental Table 3.** Relative abundance of identified metabolites. Relative abundances calculated from positive and negative ion modes. FDR corrected *p*-values for known metabolites between term and pre-term fecal samples.

**Additional file 4: Supplemental Table 4.** Enriched pre-term metabolic pathways mapped to *Escherichia coli*. A list of microbial metabolic pathways enriched in pre-term stool samples compared to term stool samples as determined by KEGG orthologs.

**Additional file 5: Supplemental Table 5.** Enriched pre-term metabolic pathways mapped to *Homo sapiens.* A list of human metabolic pathways enriched in pre-term stool samples compared to term stool samples as determined by KEGG orthologs.

## Data Availability

16S rRNA sequences have been uploaded to the National Center for Biotechnology Information Sequence Read Archive (NCBI SRA) under BioProject ID: PRJNA533302. All data generated or analyzed from global untargeted LC-MS are included in this published article and its supplementary information files. All other datasets used and/or analysed during the current study are available from the corresponding author on reasonable request.

## Abbreviations

ASV: Amplicon sequence variant
DCA: Deoxycholic acid
EGF: Epidermal growth factor
EGFR: Epidermal growth factor receptor
FXR: Farnesoid X receptor
GDCA: Glycodeoxycholic acid
KEGG: Kyoto Encyclopedia of Genes and Genomes
LAB: Lactic acid bacteria
LefSe: Linear discriminant analysis effect size
LC-MS: Liquid chromatography-mass spectrometry
NCBI: National Center for Biotechnology Information
NEC: Necrotizing enterocolitis
PC: Principle component
PCA: Principle component analysis
PCoA: Principal coordinate analysis
PERMANOVA: Permutational multivariate analysis of variance
PGE2: Prostaglandin E2
SRA: Sequence read archive
TCA: Tricarboxylic acid cycle
TUDCA: Tauroursodeoxycholic acid
UHPLC: Ultra-high-performance liquid chromatography
